# Techniques and countermeasures for preventing insider threats

**DOI:** 10.7717/peerj-cs.938

**Published:** 2022-04-01

**Authors:** Rakan A. Alsowail, Taher Al-Shehari

**Affiliations:** Computer Skills, Self-Development Department, Deanship of Common First Year, King Saud University, Riyadh, Saudi Arabia

**Keywords:** Security and privacy, Insider threat prevention, Theoretical and empirical aspects, Rigorous literature review

## Abstract

With the wide use of technologies nowadays, various security issues have emerged. Public and private sectors are both spending a large portion of their budget to protect the confidentiality, integrity, and availability of their data from possible attacks. Among these attacks are insider attacks which are more serious than external attacks, as insiders are authorized users who have legitimate access to sensitive assets of an organization. As a result, several studies exist in the literature aimed to develop techniques and tools to detect and prevent various types of insider threats. This article reviews different techniques and countermeasures that are proposed to prevent insider attacks. A unified classification model is proposed to classify the insider threat prevention approaches into two categories (biometric-based and asset-based metric). The biometric-based category is also classified into (physiological, behavioral and physical), while the asset metric-based category is also classified into (host, network and combined). This classification systematizes the reviewed approaches that are validated with empirical results utilizing the grounded theory method for rigorous literature review. Additionally, the article compares and discusses significant theoretical and empirical factors that play a key role in the effectiveness of insider threat prevention approaches (e.g., datasets, feature domains, classification algorithms, evaluation metrics, real-world simulation, stability and scalability, *etc.*). Major challenges are also highlighted which need to be considered when deploying real-world insider threat prevention systems. Some research gaps and recommendations are also presented for future research directions.

## Introduction

Due to the spread use of technologies in the last decades, issues of security and privacy have been extremely increased. Organizations are holding sensitive assets (*e.g.*, customer data, business plans, intellectual properties, *etc.*), which could cause a huge damage to their business and reputation, if they have been breached. Therefore, it is of great importance to all organizations to protect the confidentiality, integrity, and availability of their sensitive assets from insider attacks. One of the major concerns in the field of information security is the insider attacks (Yaseen & Panda, 2012), as they were reported to be the most common attack in 2017 with around 60% (Lee et al., 2020).

### Insider threats

The insider threats are malicious acts that are carried out by authorized persons, which may cause detrimental implications for digital and physical assets of an organization. In Sinclair & Smith (2008) an insider is defined as “any person who has some legitimate privileged access to internal digital resources, *i.e.,* anyone who is allowed to see or change the organization’s computer settings, data, or programs in a way that arbitrary members of the public may not. This includes full-time employees, but may also include temporary workers, volunteers, and contractors, depending on the nature of the business”. The Computer and Emergency and Response Team (CERT) emphasized the malicious intention of the insider by defining the insider as “a current or former employee, contractor, or business partner who has or had authorized access to an organization’s network, system, or data and intentionally exceeded or misused that access in a manner that negatively affected the confidentiality, integrity, or availability of the organization’s information or information systems” (Claycomb & Nicoll, 2012).

### Insider threat incidents and their impacts

Whether malicious acts of insiders were intentional or unintentional, they can cause an equally harmful impact, such as stealing, leaking and damaging sensitive data, or even helping external attackers by creating backdoors for them to attack. The severity of attacks caused by insiders can be noticed from the following examples of occurred real-world incidents (Hunker & Probst, 2011). The first example, a serious insider attack which destroyed the image of both the Federal Bureau of Investigation (FPI) and the U.S. was conducted by an employee of the U.S. National Security who leaked high confidential data to Russian agencies. Another insider attack was carried out by a soldier of the U.S. army who leaked huge highly classified government documents to WikiLeaks. Moreover, the most serious fraud incident, which cost the Societe Generale French bank an estimated amount of $7 billion, was conducted by one of its employees.

In addition, 1,154 actual insider threat incidents in Collins (2016) were reported by the U.S. Security Service and CERT. Such insider attack incidents have been classified into different categories: sabotage, fraud, theft, and miscellaneous. A number of 659 from the reported incidents fell under the category of fraud in which data were modified or deleted for the aim of personal gain, whereas 189 of the reported incidents fell under the category of theft, where intellectual properties of organizations were stolen. The rest of the reported incidents fell under the categories of sabotage and miscellaneous, where the aim was to disrupt business operations of organizations. While some organizations have reported the occurred insider attack incidents, other organizations have not. This is because they are afraid of the negative impact that may face if the executed insider attack incidents are announced to the public (Roy Sarkar, 2010).

### Preventing insider threats

The reliance on the utilization of digital assets presents a real challenge on how to secure them. Such assets exist within the boundaries of the organizations in PCs, USB devices, emails, memo and networks. Securing such sensitive digital assets is of great importance to the continuity and advancement of organizations. To prevent insider threats, some companies have taken drastic measures, such as employee vetting, authentication mechanisms, training, surveillance, separation of duty, and so on Erdin et al. (2018). Insider threats are the most challenging to detect, and traditional techniques cannot easily mitigate them Almehmadi (2018).

A large number of works in the literature focused on detecting and preventing insider attacks. CERT has contributed widely in such work by providing periodic guidelines that include the best practices for insider attack mitigation (Silowash et al., 2012). Different approaches for protecting against insider threats can be categorized into three classes (detection approaches, detection & prevention approaches, or prevention approaches). *In the first class*, insider threats are detected during or after the threat has happened. *In the second class*, insider threats are detected and then they are prevented but while or after some parts of the threats are happening. *In the third class*, insider threats are prevented before they are carried out. The third class is the optimal solution for insider threat prevention but the hardest to achieve. It is noticed that most of the existing approaches focused on the first class “detection approaches”, such as in Alsowail & Al-Shehari (2020), Roberts et al. (2016), Chen, Nyemba & Malin (2012), Gates et al. (2014), Axelrad et al. (2013), Legg et al. (2017), Raissi-Dehkordi & Carr (2011) and Parveen et al. (2011). More insider threat detection approaches can be found in Bertacchini & Fierens (2009), Ben Salem, Hershkop & Stolfo (2008), Zeadally et al. (2012), Gheyas & Abdallah (2016) and Ko et al. (2017).

The huge damage caused by successful insider attacks to many organizations have made it crucial to prevent such attacks. In our research of interest, we conducted a thorough search to figure out the research gaps in the insider threat prevention area which are not addressed yet. As a result, we found that there are two surveys (Cheng, Liu & Yao, 2017; Liu et al., 2018) that reviewed the insider threat prevention approaches from limited perspectives. The survey in Cheng, Liu & Yao (2017) focused on data leakage detection and prevention techniques. It classified them into two categories: content-based approaches and context-based approaches. It highlighted, in a too summarized way, some technical challenges for data leak detection that still need to be addressed (*e.g.*, Scalability, Privacy Preservation, Accuracy and Timeliness). The survey in Liu et al. (2018) focused on insider threat detection and prevention techniques from a data analytic perspective. It categorized the relevant studies into host, network, or contextual audit data source, such as how the data are extracted and what are the utilized algorithms. However, we found that a review that explores and discusses the main characteristics of the insider threat prevention approaches (*e.g.*, prevention methods, datasets, features domain, algorithms, tools, accuracy metrics, *etc.*) is missing in the literature. Therefore, as different from existing surveys, our study reviews and discusses the insider threat prevention approaches by classifying them into major categories (biometrics, asset-metrics, *etc.*). Then, it discusses and compares them from different theoretical and empirical aspects. This survey will serve as a guide for future researchers to observe insider threat prevention body of knowledge from various prospective. The proposed classification model, discussed empirical and conceptual factors, and highlighted research challenges will provide the insider threat research community with updated review for devising more effective insider threat prevention. The key contributions of this article are summarized as follows:

1. A unified classification model is proposed to classify the insider threat prevention approaches into two categories (biometric and asset-metric). The biometric-based category is also classified into (physiological, behavioral and physical), while the asset metric-based category is also classified into (host, network and combined). Such classification model systematizes the insider threat prevention approaches based on the major factors that play a key role in insider threat prevention contexts.
2. It discusses some significant factors (theoretical and empirical) that affect the performance and the scope of insider threat prevention approaches as follows: detection and prevention *vs* detection, behavioral *vs* physiological, simulating real-world situations, human factor interventions within automated processes of a solution, scalability of an approach, demonstrating experimental setting details, datasets, feature domains, classification algorithms, evaluation metrics and the stability of obtained results over time. Thus, we deem that such factors are crucial and should be taken into consideration when developing and implementing insider threat prevention systems.
3. It presents some challenges of deploying real-world insider threat prevention systems. Such challenges are still an open challenge; therefore, they are discussed in terms of how they can be addressed in the future. Moreover, some recommendations are also presented according to lessons learned from reviewed approaches.

The remainder of the article is organized as follows: The applied research methodology is summarized in ‘Survey Methodology’. Our classification model is illustrated in ‘Classification Model’. The theoretical and empirical factors and observations are discussed in ‘Observations, Discussions and Recommendations’. Industrial insider risk management tools are summarized in ‘Insider Risk Management Tools (IRMT)’. Some research challenges and recommendations are highlighted in ‘Research Challenges’. Finally, the ‘Conclusion’ concludes this work.

## Survey Methodology

To achieve the contributions of this article, we applied the grounded theory (Wolfswinkel, Furtmueller & C. Wilderom, 2013) as it is well-known methodology for rigorous literature review. It has been utilized widely to analyze research topics for building theories based on observations and findings from reviewed articles. The five stages of this methodology are presented in Table 1.

**Table 1:** Five stages of grounded theory.

Stage	Task
1. Define	1.1 Define criteria for inclusion or exclusion
	1.2 Identify the field of the research
	1.3 Determine appropriate academic sources
	1.4 Decide specific searched keywords
2. Search	2.1 Search
3. Select	3.1 Refine the downloaded articles
4. Analyze	4.1 Open coding
	4.2 Axial coding
	4.3 Selective coding
5. Present	5.1 Represent and arrange the content
	5.2 Structure the article

Starting the work with a well-defined topic allows us to review and analyze selected articles thoroughly. In this section, we summarize the applied methodology as follows:

**Figure 1:**
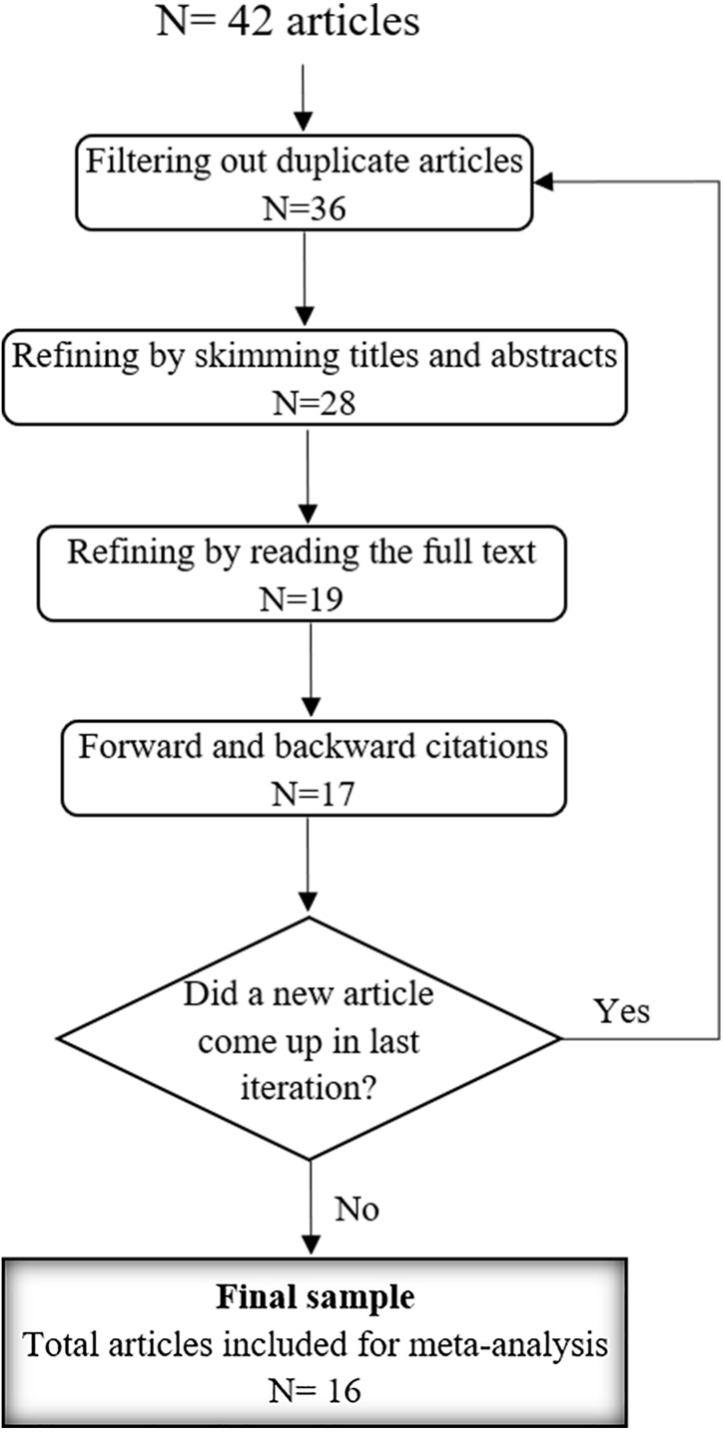
The strategy utilized for selecting the articles based on the Ground Theory.

1. Define: In the initial stage, the scope of the research topic is defined. As insider threat area is quite broad, we specify the studied topic as (insider threat prevention approaches that are validated with empirical results). Concerning this, theoretical approaches are beyond the scope of this study. Next, the criteria for inclusion and exclusion of articles are identified. This is by specifying searched keywords as (insider threat prevention and preventing insider threat). The appropriate academic sources are also identified (Web of Science, Google Scholar and Scopus).
2. Search: In the second stage, utilizing specified criteria we search for target articles. While searching, the synonyms of searched keywords are taken into account to ensure that we cover the entire scope of the topic. This is done by searching for the keywords (*e.g.*, insider threat, insider attack, insider prevention, preventing insider attack/threat) utilizing AND/OR operators interchangeably. Furthermore, the search keywords are typed in the search box of academic sources within quotes in order to narrow down and refine the obtained results. The acquired articles are selected from journals and conference proceedings. Additionally, we refer to the bibliography of previous works to make sure that we have not left out any relevant work. At the end of this stage, we obtained 38 articles.
3. Select: In this stage, the refining process of downloaded articles is performed as depicted in Fig. 1. The pre-selection of articles is determined by skimming titles, abstracts and pertinent sections of the articles. Thereafter, we read the full text of the articles. After excluding duplicate and irrelevant articles, we select the articles that are published in leading journals and conference proceedings according to the Web of Science Core Collection (Collection, 2013). At the end of the selection stage, we ended up with 16 articles as shown in Fig. 1.
4. Analyze: In this stage, the crucial part of implementing Grounded Theory is applied, *i.e.,* the selected articles are categorized and analyzed. While reading each article carefully, the related concepts, findings and insights are highlighted for further analysis. This is accomplished by implementing the coding processes (open coding, axial coding and selective coding) of grounded theory (Wolfswinkel, Furtmueller & C. Wilderom, 2013). In open coding, high level categories are produced based on highlighted concepts and findings. The relation between categories and subcategories are made in axial coding. Then, the categories and subcategories are joined and refined in the selective coding process. To carry out these processes, we employ the Saturate tool (www.saturateapp.com), a web-based open coding tool that allows for code-to-data traceability.
5. Present: In the last stage, the insider threat prevention approaches are categorized according to our classification model. Furthermore, the observations and discussion of proposed factors are presented in ‘Observations, Discussions and Recommendations’.

## Classification Model

**Figure 2:**
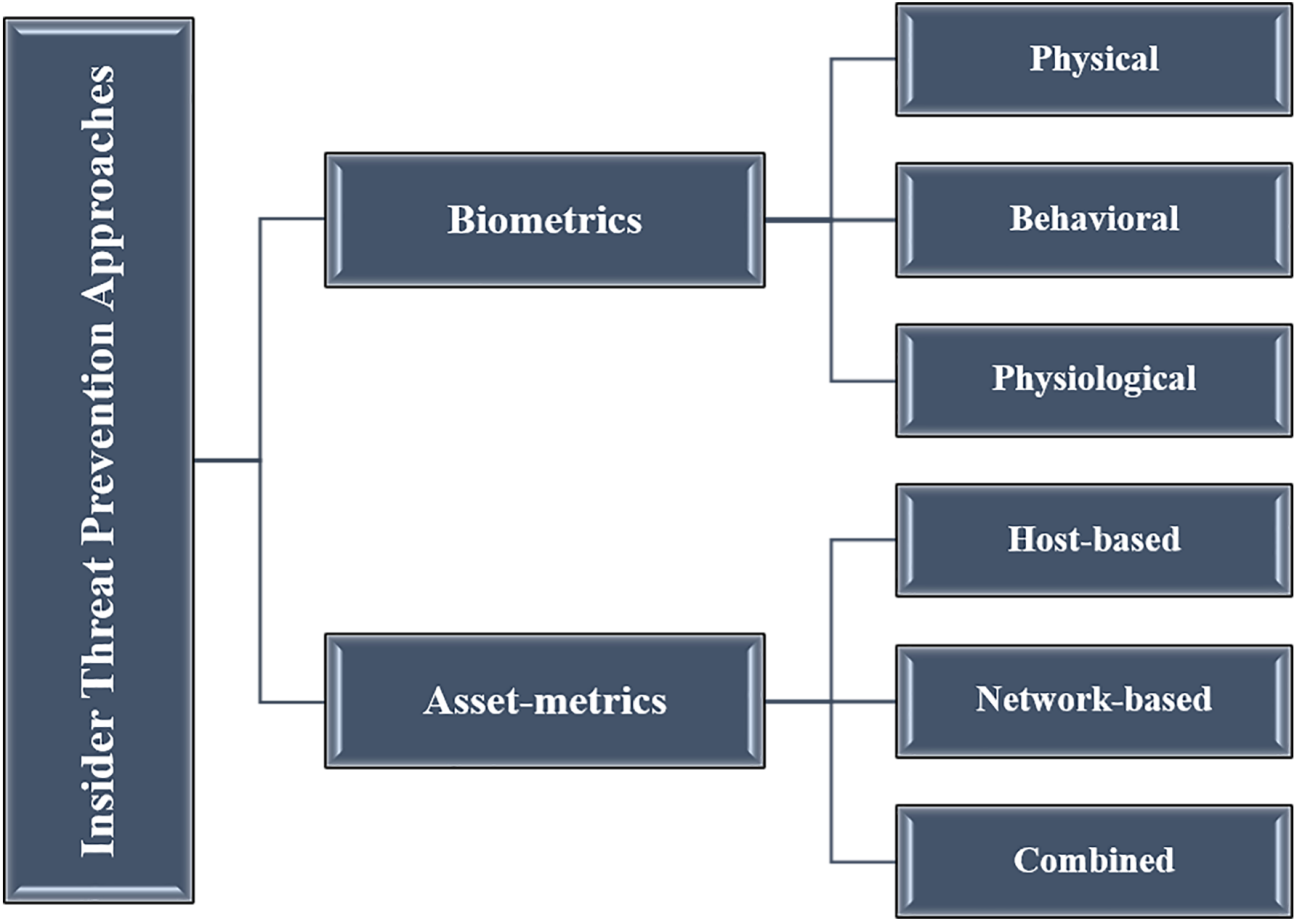
The proposed classification model of the insider threat prevention approaches.

As mentioned above, tremendous losses have been incurred due to the rising number of insider attacks. As a result, various solution approaches have been introduced in the literature, most of them are focused on the detection approach “how to detect insider attacks” which have been reviewed in Bertacchini & Fierens (2009), Ben Salem, Hershkop & Stolfo (2008), Zeadally et al. (2012), Gheyas & Abdallah (2016) and Ko et al. (2017). However, in this article we review existing works that are focused on prevention approaches “how to prevent insider attacks”. In this section we demonstrate our classification model as depicted in Fig. 2.

### Biometric-based

The reality is that insider threats are human-based, and hence should be dealt with by employing biometrics. Biometric technology is the analysis of a person’s physical, physiological or behavioral features (Jain, Ross & Pankanti, 2006). A number of approaches, illustrated in the subsequent sections, have been applied to validate legitimate users from fraudsters. Some strategies have made use of the brain signals, typing behaviors, eye movements, and body movements of insiders for the aim of preventing insider threats.

#### Physical biometrics

Applying human-based characteristics (biometric measurements) in the field of information security has been an active area of research for many years. It has continuously evolved from physical/hard biometrics (*e.g.*, fingerprints, eye iris, and facial patterns) to physiological biometrics (*e.g.*, brain signals). Physical biometrics enable the discrimination between individuals with high accuracy rate, which cannot usually be changed during the lifetime of a person (Eberz et al., 2016). However, although physical biometrics is hard to be mimicked, it can still be exploited by attackers due to the high-level advancements in technology gadgets. For example, Barral & Tria (2009) showed that fingerprint sensors can be attacked using mock fingers. In addition, Boehm et al. (2014) presented that a facial recognition attack is possible using complex 3D video software.

It is worth noting that physical biometrics are usually used to verify users before granting them an access to an organization asset. In insider threat literature, we have not found a study that implemented physical biometrics to prevent “masquerader” attack, a person who may acquire an access to the PC of his/her colleague illegitimately and conduct malicious acts. In other words, if an insider login to his/her PC and leaves without signing out from the session, a masquerader attack can occur. Thus, a research gap that can be bridged here to prevent this type of insider attacks. This can be achieved by developing a continuous authentication mechanism by utilizing physical biometrics (*e.g.*, eye iris or facial patterns) to verify insiders throughout their sessions continuously.

#### Behavioral biometrics

Various biometrics have been used to improve the protection against insider attacks. Behavioral biometric was introduced by some of the reviewed approaches (*e.g.*, typing patterns, head and eye motions). One science of biometric is Keystroke dynamics, where insiders, based on their typing habit, are authenticated constantly. Babu & Bhanu (2015) introduced an approach which aims at detecting and preventing masqueraders’ attacks by integrating typing patterns of insiders with an access control model. The model is made up of two phases. Risk scores are linked to resources using Common Vulnerability Scoring System (CVSS) in the first phase, and continuous validation of insider typing is tracked during the whole session (using keyloggers) in the second phase. The Support Vector Machine (SVM), as a classifier, and CERT insider threat database were both utilized to conduct the simulation testing. The variations between presses and releases of insider keystroke patterns were calculated. Once an anomalous typing pattern is detected, the tasks in execution will immediately be blocked, as considered a masquerader attack. The risks in the model are categorized into low, medium, high and critical, and results are presented for different scenarios.

In Jeong & Zo (2021), a research was conducted to study the impact of opportunity-reducing called as hard form techniques (*e.g.*, perceptions and behavioral intentions) that lead users in disrespecting the organizational security policies. They are labeled as opportunity regulations which are useful for handling insider risks. A scenario-based questionnaire survey was carried out on 5,158 members who have a working experience of services in different fields (*e.g.*, industry, research and development). Utilizing the least square based structural equation modeling (PLS-SEM), 259 responses were analyzed. The study showed that hard controls might weaken the relationships between users and their organizations. The results show that the hard techniques make users feel that their privacy is intruded which lead them to be less hesitant for committing insider attacks as well as making their behavior to be morally irresponsible. Thus, the study recommended that a harmonic integration of soft and hard controls should be utilized. It also suggested that strategic options and tactics that can ease and enable soft-landing of hard controls are necessary for coping with insider threats and decision-making processes for security policies. The authors presented a limitation for their study in that it has not determined in what way to make users observe hard controls as less invasive. The study emphasized the need for a detailed exploration for organizational security policies to overcome the limitation in future research.

Another approach to predict the intention of insider access based on behavioral biometrics by monitoring head micro-movements was introduced by Almehmadi (2018). Two scenarios (accessing protected files and burning a lab) performed by 40 participants are used to perform the experiments. The head movements of participants were monitored by mounting gyro sensors on their heads in order to predict their intention of access. The collected data is immediately analyzed by Testbench–Emotiv EEG software. According to the testing results, there was a correlation between the number of insiders’ head micro-movements and the motivation toward executing intentions. The author found out that the probability of executing insiders’ intentions is increased when their head micro-movements are lower. The findings indicate that with an accuracy of 70%, the approach is able to prevent insiders’ malicious acts.

Furthermore, a behavioral approach that is based on eye motion features was proposed by Eberz et al. (2016) to authenticate users. Their focus was on masqueraders’ threat, and to develop a constant authentication system utilizing users’ gaze features in order to differentiate them from each other. An experiment with 30 participants (20 males and 10 females) was performed to assess the applicability of the approach. By using the SMI RED500 eye tracking device, the gazing features of the participants were collected during tasks they were asked to perform. A number of 21 gaze features were extracted (*e.g.*, pupil diameters, temporal, spatial, *etc.*). The scenarios, where the participants were wearing eye glasses or contact lenses, were also tested. k-Nearest-Neighbors (kNN) and Support Vector Machine (SVM) were utilized for the classification. Five times the experiments were repeated with different scenarios, and the approach accuracy was estimated at 84.56%.

#### Physiological biometrics

The main goal of access control models is to regulate access to digital assets through various authentication methods, *e.g.*, passwords, tokens, fingerprints, *etc.*, so that access can only be granted to authorized users with the right permissions. A major problem of access control models in general is that once a user has been granted access to a digital asset, the user will be trusted throughout the session (Almehmadi & El-Khatib, 2017). Hence, the user will be able to misuse the granted privileges without being detected. To overcome this problem, Intent-Based Access control Model (IBAC) was proposed. Unlike traditional access control models, IBAC verifies the integrity of insiders’ intent rather than their identities. The idea of IBAC is that physiological features, such as brain signals, can be utilized to detect the honesty of intentions for preventing insider threats, since such threats are human-based.

In our classification model, we categorize this type of approach under physiological biometrics. Almehmadi & El-Khatib (2017) proposed the implementation of IBAC in the field of insider threats. The authors utilized brain signal features to detect intentions of insiders, and then determine whether to grant them access to organizations assets or not. Based on validated knowledge of insiders’ motivation, a risk level is calculated. Then, granting access to an asset or not is determined by a threshold of risk level. The risk level is calculated based on the brain signals amplitude to evaluate the probability of executing the intention of insiders. A couple of experiments of 30 participants are performed to validate the approach. The experiments have simulated two malicious intentions of insiders (opening secured files and burning physical resources). The two main bases of IBAC techniques, P300-based concealed information test (CIT) and brain-computer interface (BCI), were utilized to detect intentions of participants. Additionally, the collection process of participants’ brain signal responses was accomplished through an Emotiv EPOC, a wireless 14-channel electroencephalogram (EEG) acquisition device. The EEGLAB—Open Source Matlab Toolbox for Electrophysiological Research was used to analyze the collected data (Brunner, Delorme & Makeig, 2013). The accuracy results of this approach achieved 100% by using the Support Vector machine (SVM) classifier. However, as it is considered the first IBAC method applied in the area of preventing insider threats, the authors recommended further research to be deployed in real life. The taxonomy of behavioral-based and physiological-based prevention approaches utilizing the biometrics of insiders are summarized in Table 2.

As presented in Table 2, the focus was to address both masqueraders and malicious insiders utilizing various behavioral biometrics (typing patterns, head and eye motions). They also applied various classification algorithms and reported different accuracy results. To a certain extent, the utilized biometrics, head micro-movements in Almehmadi (2018) and eye motions in Eberz et al. (2016), showed almost high accuracy of 92.2% and 84.56%, respectively. Such works reveal a new trend to correlate multiple types of biometrics to prevent insider threats with high accuracy. With respect to eye-tracking technologies, they have been implemented in several areas, such as Eberz et al. (2016), Rayner et al. (2001) and Meißner & Oll (2019). Such eye-tracking techniques can give insights to be employed for preventing masquerader threats.

**Table 2:** Biometric-based approaches.

Approach	Addressed threat	Feature domain	Dataset	Classification technique	Accuracy	Ref.
Behavioral	Masquerader	Typing patterns	CERT	SVM	Misc.	Babu & Bhanu (2015)
Behavioral	Malicious Insider	Head micro- movements	Synthetic	NA	92.2%	Almehmadi (2018)
Behavioral	Masquerader	Eyes motions	Synthetic	kNN & SVM	84.56%	Eber et al. (2016)
Behavioral	Malicious Insider	perceptions and behavioral intentions	Survey instruments	PLS-SEM	Misc.	Jeong & Zo (2021)
Physiological	Malicious Insider	Brain signals	Synthetic	SVM	100%	Almehmadi & K. El-Khatib (2017)

With regard to physiological-based approach (Almehmadi & El-Khatib, 2017), even though it achieves brilliant results, there is a need to improve its deployment, acceptability, and scalability as follows: (a) The deployment of the approach relies on brain signals, and hence, it can be affected by external factors that may distort the results obtained; (b) Regarding scalability, the current implementation of the approach is suitable for a small number of malicious intents, however, it will be more complicated to protect against a large number of malicious intents. The current approach only considers two types of malicious intents, which are opening secured files and damaging physical assets, while IBAC should rely on enormous categories of intents to be more effective. Scalability can be improved by integrating a role-based access control model (RBAC) with the IBAC model. Gaining obvious knowledge of roles and authorization of insiders, can eliminate the need for huge categories of insiders’ intents while achieving high accuracy of risk levels; (c) Another issue is the acceptability of the approach in actual environments. For implementing this approach, an organization needs to mount the sensors of brain signals on the heads of their insiders (*i.e.,* employees). Therefore, such practice will not satisfy the insiders, and enforcing it might reduce the trust and the productivity in the work environment.

Despite the fact that IBAC physiological-based techniques presented promising results, as presented in Almehmadi & El-Khatib (2017), an improvement is needed to overcome the limitation of the current approach particularly in the aspects of deployment, scalability and acceptability.

### Asset-based metrics

We have described above two approaches which are behavioral and physiological biometrics. In this section, we present the asset-based approaches which are categorized into host, network and combined.

#### Host-Based

The initial research in the field of protection against insider threat focused on preventing malicious acts at the database application level. Chagarlamudi, Panda & Hu (2009) suggested a model focused on particular tasks and transactions to avoid malicious database acts. Petri nets, a directed bipartite graph that consists of nodes and transitions, were utilized to validate the model (Murata, 1989). In the experiment, two parameters (normal and malicious tasks) were simulated to demonstrate how the graphical modeling can be used to prevent unauthorized data modifications. In their study, they found that false negatives can reach as high as 100% for a single transaction task, while they can go as low as 0% for a five-transaction task. Evidently, the false negative rate increases when the number of transactions per task increases.

Furthermore, the authors in Ragavan & Panda (2013) proposed an approach to prevent unauthorized data modification on a database, by attaching a variable named “threshold” with every single data item on the database. The threshold determines the maximum degree to which a data item can be changed. As a result, any update operations on the data item that surpassed the threshold will be blocked. Two models were utilized in the experiment (log entries and dependency graphs). Five thousand data items were traversed by the models, which used a range of parameters (*e.g.*, number of data items, transactions, and dependencies). The authors were more concerned with the performance of their approach than its accuracy. They revealed that the monitoring changes for each object in a large database creates delays and slows down the system. To resolve this performance problem, they classified and labeled each item on the database based on its value to the organization. Thus, the priority will be given to Critical Data Items (CDI) for preventing any malicious update on them. Based on the results, the dependency graph model detects malicious operations faster than the log entry model on various scenarios.

Insiders can carry out a data leakage attack, which could have significant ramifications for organizations. A hybrid Data Leak Prevention framework was proposed by Costante et al. (2016) in order to prevent this type of attack. Two engines in their framework were combined, signature-based and anomaly-based. The framework tracks insiders’ acts in order to detect possible anomalous transactions. The anomaly-based engine then notifies the security operator, who checks whether or not a detected transaction was malicious. Following that, the framework creates signatures, which are used to prevent similar transactions from being executed in the future. Both synthetic and real-world datasets were used to test the framework. The synthetic dataset was created using data from a healthcare management system that included 30,490 SQL transactions over a period of 15 days. The actual dataset consisted of 12,040,910 SQL transactions extracted from an Oracle database belonging to a major Dutch IT firm. The findings revealed a range of a false positive rate of preventing data leakage threats.

Insiders can easily exploit USB ports that exist in most computers used today. To overcome USB malicious code attacks, the authors in Erdin et al. (2018) introduced a hardware-based scheme. In their experiment, the attack scenario involved insiders who inserted malicious code into the PCs of their colleagues *via* USB devices. The scheme was evaluated on the ZedBoard (Louise H Crockett & Elliot, 2015), a USB development board, where USB packets can be customized and tracked using a Logic Analyzer. As a result, USB packets are used to gather information about USB devices (*e.g.*, vendor IDs, device IDs, number of endpoints, type of endpoints, *etc.*). Descriptors are specified *via* USB configuration input to prevent possible malicious code attacks. The experiment was implemented on various OS platforms, including Linux and Windows, to check the independence of their scheme’s hardware.

In Lehrfeld (2020), a model was proposed to prevent intellectual property leak through USB devices. This is by enabling an organization to adapt its security access control by blocking USB write and allowing read-access capability. So, the users throughout the organization can read data from their USB devices, but the exfiltration of data they can’t. The model is implemented in USB drivers of virtual machines. A USB write-blocking script is utilized to simulate a malicious insider who can copy files from virtual machines into USB devices. The results show that the model was successful in blocking the intellectual property leaks with an accuracy over 90%. However, the authors presented some limitations of the model. They indicated that the implementation of the model in an enterprise is out of scope of this study. Also, the implemented script can’t be run in an anonymous way, so that it is not reflecting the real-world scenario of an insider intellectual property leak. In addition, a user with admin access privileges can disable or pause the running of the script on USB ports which stops the script from execution and enables the copy of files to USB devices. The authors discussed a wide array of options which can be accomplished for enhancing the proposed model in future research directions.

A freeware Data Leakage Prevention (DLP) system (Thombre, 2020) was proposed to protect sensitive data in small and medium scale organizations. Although there are several channels for exfiltrating data (*e.g.*, E-mail, Bluetooth, *etc.*), the USB is the most well-known channel for data transfer. So, the proposed DLP system is designed for the windows platform to prevent the transferring of confidential files through USB ports. The system is designed to monitor the move and copy operations that are conducted from a PC to any USB devices continuously. This can be done based on security policies and criteria that can be set by system administrators. For the aim of introducing a novel data leakage prevention solution, the proposed system leverages kernel space modules and machine learning for checking the contents of transferred files and blocking file transfer actions in case of confidential files.

#### Network-based

The spread of computer networking nowadays has raised many challenges. In particular, preventing data leakage threats of insiders, who may have privileges over the organization systems or networks. The characteristics of network traffic patterns have been utilized in many subjects of information security and privacy, such as in Al-Shehari & Zhioua (2018). For preventing insider threats over the network, the authors in Sibai & Menascé (2011) proposed the Autonomic Violation Prevention System (AVPS). It is an extension to their previous work in Sibai & Menasce (2011) that was concerned with the scalability of their approach. In their proposed system, access to a network is limited and controlled *via* in-line components that monitor the act of an insider on a network. Then, the insider act is taken based on associated conditions with incidents of data leakage threats. This was accomplished by the use of Event-Condition-Action (ECA) autonomic policies (Huebscher & McCann, 2008), which are widely used in security-centric systems. Several tests were carried out to evaluate the performance of their system across a variety of network applications (*e.g.*, FTP, database, and Web servers). The tests were conducted on RedHat, Ubuntu Linux, and Fedora operating systems. Snort was used to process network traffic packets and extract attributes (*e.g.*, IP, user, application type, request, response, *etc.*). The information gathered was analyzed and normalized before being compared to policies and rules. When a breach is detected, an action is taken to prevent malicious transfers. The efficiency was assessed using three metrics: throughput, CPU consumption, and transfer time, all of which had 95% confidence intervals.

#### Combined

Since insiders have permissions to use a variety of organization resources, various attributes can be utilized to prevent possible malicious acts. The widespread use of mobile devices and social media presents an opportunity to be incorporated into protection systems. Obtaining geo-context information of insiders related to their work environments can help to detect suspicious insiders and hence prevent associated threats. Moreover, granting or denying access to an organization asset can be determined through such information (Eberz et al., 2016). For example, an insider who constantly stands in positions where he/she is not supposed to be in should be flagged as suspicious and denied from accessing high-value assets by an ideal security system. Concerning this, in Baracaldo, Palanisamy & Joshi (2019) a Resilient Access Control Framework (G-SIR) was proposed to detect the trustworthiness of insiders before granting them an access to specific assets. Current and historical geo-social information of insiders, including social networks that were represented as social graphs and user mobility that was represented as locations on maps, are linked for access control decisions by the framework. The authors in O’Madadhain et al. (2005) validated the framework by creating synthetic dataset using Jung API. The stability of the framework was confirmed by using 250 insiders and repeated 30 times. The approach was able to prevent insider attacks with an accuracy rate of 76%.

In Liu et al. (2020), a hybrid framework for intellectual property theft detection and prevention was proposed. It integrates a prevention module with an anomaly detection module. The prevention module utilized a blacklist mechanism for preventing known insider attacks through applying two phases (the prevention phase and the blacklist management phase). In the prevention phase, an insider activity is matched against a blacklist, so if it is included within the blacklist, the insider’s act will be blocked and all homologous activities will be blocked as well. Otherwise, it is passed to the detection module for verifying whether it matches the previously known normal act or not. This is used for updating the profile of the normal activities model utilizing an operator who is responsible for analyzing the raised alert. So, if the alert is recognized as a false positive, the normal activities profile is updated, otherwise, it is identified as a malicious act. The decision to append it to the blacklist was based on the analysis knowledge of the operator. The experimental results showed that the framework can reduce the efforts of the operator by preventing insider acts within a time of around 0.5 Ms. The framework can also reduce the spread of intellectual property leakages as well as and the damages that may cause.

This section discusses the insider threat prevention approaches that are based on asset-metrics (host, network and combined). A summary of their factors (addressed threat, feature domain, dataset, classification technique and evaluation metrics) are compared in Table 3.

**Table 3:** A summary of the asset-metrics based approaches for preventing insider malicious acts.

Ref.	Addressed threat	Approach	Feature domain	Dataset	Classification technique	Evaluation metrics
Erdin et al. (2018)	USB malicious codes	Host-based	USB device	Synthetic	Rule matching	Transfer time, latency
Chagarlamudi, Panda & Hu (2009)	DB modifications	Host-based	DB Transactions	Synthetic	Rule matching	False Negatives
Ragavan & Panda (2013)	DB modifications	Host-based	DB transactions, and dependencies	Synthetic	Log-based & Dependency-based	Frequency and time
Costante et al. (2016)	Data leakage	Host-based	SQL queries	Synthetic & Real	Rule matching & Anomalous	False positives
Lehrfeld et al. (2020)	Intellectual Property Theft	Host-based	USB device	Synthetic	Rule matching	No. of blocked cases
Thombre (2020)	Data leakage	Host-based	USB ports	Synthetic	Rule matching	No. of blocked cases
Al-Shehari & Zhioua (2018)	Intellectual Property Theft	Combined	Files operations	Synthetic	Rule matching & Anomalous	Precision, Recall, and F-measure
Sibai & Menasce (2011)	Data leakage	Network-based	Packets traffic	Synthetic	Rule matching	Throughput, transfer time, CPU usage
O’Madadhain et al. (2005)	Suspicious v insiders	Combined	Geo-Social	Synthetic	Anomalous modeling	TP, FN, FP, TN

A recent comprehensive framework for preventing insider threats was proposed in Alsowail & Al-Shehari (2021). It analyzes three types of insider threat countermeasures: measures taken before insiders enter a company, measures taken during their working time within an organization, and measures taken after they depart an organization. Such countermeasures included technological, psychological, behavioral, and cognitive measures that lasted from before an insider joined the company until after they left. Three insider threat scenarios were used by the authors to demonstrate their approach.

## Observations, Discussions and Recommendations

**Figure 3:**
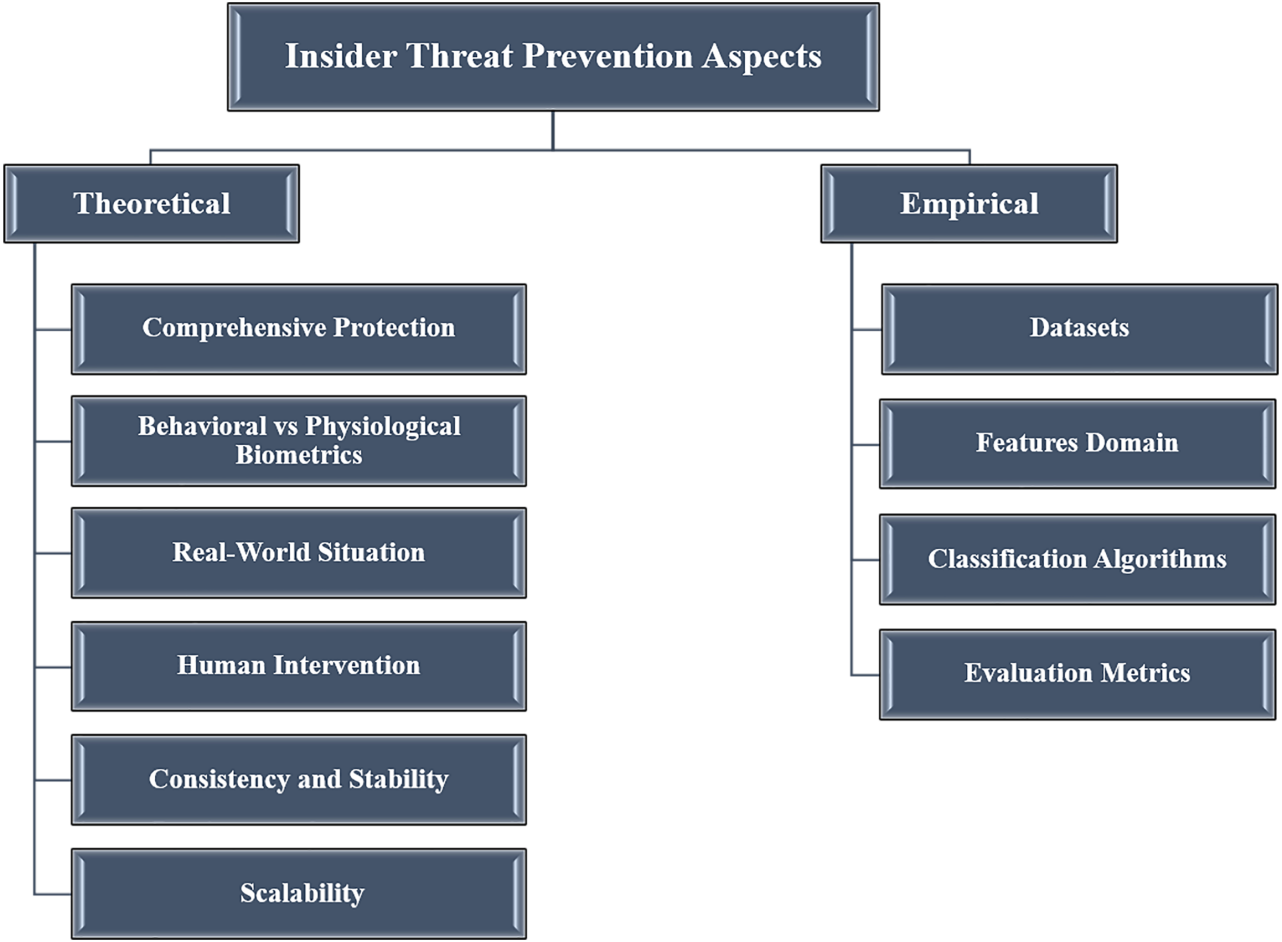
Theoretical and empirical aspects of reveied apparoches.

The aforementioned approaches employed diverse behavioral, physiological and asset metrics (*e.g.*, typing patterns, head and eye motions, brain signals, *etc.*). They also applied various mechanisms, such as datasets, feature domains, classification algorithms, accuracy and performance metrics, *etc.* The next sections discuss significant aspects (conceptual and experimental) of the reviewed works from different perspectives. The aim is to help readers and researchers to understand the applied approaches for the aim of devising more effective solutions. The discussed aspects of the insider threat prevention approaches are summarized in Fig. 3.

### Theoretical aspects

This section involves several subsections that discuss some significant factors affecting the applicability and performance of the insider threat prevention approaches, which can be clarified through the following questions:

• How is the comprehensive protection of an approach?
• Does an approach detect and then prevent an attack while occurring or it prevents an attack before occurring?
• What are the pros and cons of both behavioral and physiological biometric approaches?
• How are the real-world situations being considered in the experimental simulations?
• How are the processes of an approach independent from human interventions?
• How is the stability of the evaluation results?
• How is the coverage or scalability of an approach?

#### The comprehensive protection

**Figure 4:**
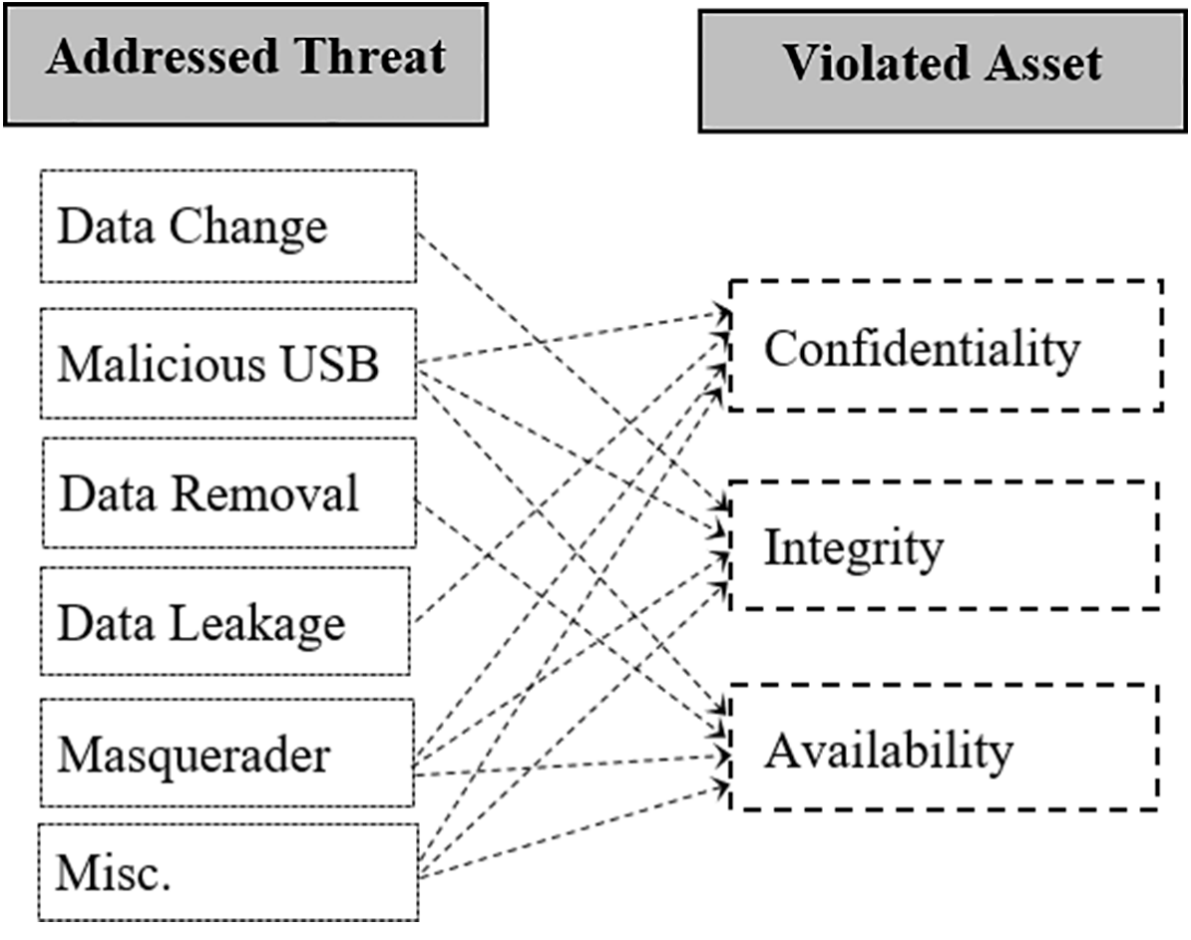
The insider threats that violate the CIA of orgnization assets.

The security goals of the data are met, if its Confidentiality, Integrity and Availability (CIA) factors are achieved. The CIA are principal factors to build any protection system. Thus, an insider threat prevention system should prevent all types of insider threats, such as data leakage, data modification, and data removal attacks, which violate the confidentiality, integrity and availability of the data, respectively. There should be an emphasis on the balance between confidentiality, integrity and availability of the data, rather than, for instance, on confidentiality alone (Olivier, 2002). So, in this section we discuss how this aspect is considered by the reviewed approaches. Figure 4 shows the insider threats that are addressed and the violation of such threats to the CIA of the data assets.

We have noticed that most of the reviewed approaches focused on protecting individual factors of the CIA and overlooked the whole protection of them at once. Table 4 shows specifically the addressed threats and protected CIA of the data asset considered by reviewed approaches. Noticeably, the approaches in Chagarlamudi, Panda & Hu (2009), Babu & Bhanu (2015), Eberz et al. (2016) and Ragavan & Panda (2013) focused on addressing data modification threats, which is fine to protect the integrity of the data. But, the protection of the confidentiality and availability of data using such approaches are still missing. In addition, the approaches in Costante et al. (2016) and Sibai & Menascé (2011) focused mainly on protecting the confidentiality of the data with regard to data leakage threats, but the integrity and availability of data are still unaddressed. On the other hand, the approaches in Almehmadi & El-Khatib (2017) and Almehmadi (2018) achieved further protection steps by protecting both the confidentiality and availability of data assets at once. Their focus was to prevent a sabotage attack of a lab (protecting the availability), and accessing secured files (protecting the confidentiality). Ideally, the balance to protect the whole CIA factors of data assets were considered by Erdin et al. (2018) and Baracaldo, Palanisamy & Joshi (2019).

**Table 4:** Addressed threats and protected Confidentiality (C), Integrity (I) and Availability (A) factors by reviewed approaches.

Ref.	Addressed threat	Insider	C	I	A
Chagarlamudi, Panda & Hu (2009)	DB modifications	Malicious		✓	
Almehmadi & K. El-Khatib (2017)	Accessing and damaging assets	Malicious	✓		✓
Babu & Bhanu (2015)	Data modifications	Masquerader		✓	
Eberz et al. (2016)	Data modifications	Masquerader		✓	
Ragavan & Panda (2013)	DB modifications	Malicious		✓	
Erdin et al. (2018)	USB malicious code injections	Misc.	✓	✓	✓
Costante et al. (2016)	Data leakages	Malicious	✓		
Sibai & Menasce (2011)	Data leakages	Malicious	✓		
Almehmadi (2018)	Accessing and damaging assets	Malicious	✓		✓
O’Madadhain et al. (2005)	Misc.	Malicious	✓	✓	✓

#### Detection *vs* prevention

The discrimination between the detection and prevention process should be highlighted, especially in the information security context. For instance, high confidential data can be seen, leaked, copied or deleted before detecting or preventing them. In this section, we categorize the reviewed approaches into “detection and prevention” or “prevention”. In detection and prevention approaches, an insider attack was detected and then it was prevented but after or while some part of the attack occurred. In contrast, a prevention approach prevents an insider attack before occurring. Table 5 shows the classification of the reviewed approaches accordingly.

**Table 5:** The classification of reviewed approaches as “detection & prevention” or “prevention”.

Ref.	Approach	Description
Chagarlamudi, Panda & Hu (2009)	Prevention	The technique prevents malicious modifications on a database by matching malicious database transactions of an insider based on predefined rules.
Almehmadi & K. El-Khatib (2017)	Prevention	The technique observes the insider’s intention of access utilizing brain signal biometrics. If there is a malicious intent, the access to an asset was prevented before an attack occurred.
Babu & Bhanu (2015)	Detection & Prevention	The technique detects a masquerader by detecting his/her anomalous typing patterns and then an attack is prevented.
Eberz et al. (2016)	Detection & Prevention	The technique detects a masquerader by detecting his/her suspicious eye motions. After that, an attack was prevented.
Ragavan & Panda (2013)	Prevention	The technique prevents malicious updates on a database by matching database transactions with predefined logs and dependencies.
Erdin et al. (2018)	Prevention	The technique prevents USB malicious codes according to predefined signatures of USB operations.
Costante et al. (2016)	Detection & Prevention	The technique detects a data leakage attack on a database by detecting anomalous SQL queries, and then such an attack is prevented.
Sibai & Menasce (2011)	Prevention	The technique prevents a data leakage attack on the network level by matching packet traffic characteristics with predefined signatures.
Almehmadi (2018)	Prevention	The technique observes the insider’s intention of access utilizing suspicious head motions. If there is a malicious intent by an insider, the access to an asset was prevented.
O’Madadhain et al. (2005)	Detection & Prevention	The technique detects the anomalous behavior of insiders based on their geo-social context. If a suspicious insider reaches a threshold of a risk level, then an attack is prevented.

#### Behavioral *vs* physiological

Biometric features are utilized to prevent a wide range of insider attacks, as they are dependent on attackers’ observable actions. One of the oldest behavioral biometric methods was proposed in 1980 to identify users based on their typing patterns (Gaines et al., 1980). Since then, several techniques have been applied to authenticate users based on their physiological or behavioral characteristics. In this regard, numerous biometric-based techniques are reviewed in Kataria et al. (2013). Unlike hard biometrics (*e.g.*, eye iris and fingerprints) which cannot be changed during the lifetime of a person (Eberz et al., 2016), physiological and behavioral biometrics can be affected by various factors. In this section we discuss the pros and cons of such factors as summarized in Table 6.

**Table 6:** Physiological vs behavioral biometric-based approaches.

Ref.	Approach	Observables	Pros	Cons
Almehmadi & K. El-Khatib (2017)	Physiological	Brain signals	− High accuracy (100%)	- Not satisfied by insiders as it requires track devices to be mounted on their heads. Outside impacts may affect the results.
Babu & Bhanu (2015)	Behavioral	Typing patterns	− Reveals the real behavior, as the profiling process is unnoticed by insiders.	It addresses data modification threats, while other threats, for instance, data removal cannot be detected. It requires a time span to detect anomalous typing. Meanwhile, the data integrity may be violated.
Eberz et al. (2016)	Behavioral	Eye motions	− Continuous authentication throughout the session.	− Addresses the masquerader attack only.
Almehmadi (2018)	Behavioral	Head micro-movements	− Continuous authentication throughout the session.	Not accepted by insiders, as it requires measurement devices to be mounted on their heads. Other human body characteristics may affect the results (e.g., breathing, talking, nature movements, etc.).

With respect to realism, the behavioral biometric approaches (*e.g.*, typing patterns) are more likely to reflect the real behavior of insiders. This is due to the internal profiling process that cannot be noticed by insiders throughout the monitoring phase, which in turn, the actual behavior of them can be observed. In contrast, the approaches that are based on physiological biometrics (*e.g.*, brain signals) are more likely to be influenced by reactions of insiders. This is because such approaches require monitoring devices to be mounted on heads of insiders, such as the EEG device in Almehmadi & El-Khatib (2017) which was utilized to monitor brain signals responses of insiders.

The acceptability and applicability of applying an insider threat prevention approach are also differing on whether an approach was developed based on behavioral or physiological biometrics. The study in Almehmadi (2018) showed that the acceptance rate of applying a behavioral biometric approach (head micro-movements) was 80%, while its accuracy rate achieved 70%. In contrast, the study in Almehmadi & El-Khatib (2017) presented that the acceptance rate of applying physiological biometrics (brain signals) was only 10%, whereas its accuracy rate reached 100%. These findings indicate that implementation of physiological biometrics outperforms behavioral biometrics in terms of their accuracy for preventing insider attacks, while the behavioral biometrics exhibited a higher acceptance rate than the physiological one. The reason is that people often reject their thoughts to be monitored when physiological biometrics are applied.

However, to acquire the merit of applying a physiological biometric approach (100% accuracy), its acceptability factor could be addressed. The study in Al-Nafjan et al. (2017) presents the state-of-the-art advancements in the brain-computer interface (BCI) area. It provides various mechanisms to recognize the emotions of computer users. So, they could be employed to address the acceptability and applicability factors of applying physiological biometrics in insider threat prevention approaches.

#### Real-world simulation

To validate the insider threat prevention approaches, various experiments are conducted by applying different real-world scenarios. The participants, who simulated insiders, performed activities as they are in a real-world environment. The participants simulated different activities that might be carried out by malicious insiders. This is important, especially when the applied approach depends on biometric characteristics. It has been noticed that some approaches tried to reflect some aspects of real-world situations. For example, Eberz et al. (2016) considered some real-world simulations (*e.g.*, wearing glasses and contact lenses) while validating their approach that was based on eye motions. The physiological biometric approach in Almehmadi (2018) is also considered some aspects of simulating real-world situations in their experiments. They put all electronic devices away from participants to ensure that the collected data is not affected by external influences. They also asked the participants, prior to the experiments, to not-divulge their malicious intentions in order to simulate a real attack scenario.

In our review, we have observed that some approaches tried to simulate some real-world situations (*e.g.*, Almehmadi, 2018; Eberz et al., 2016), while a holistic simulation of preventing real-world insider attacks is still missing. Therefore, a holistic view of preventing insider threats requires further study to integrate various aspects within an organization, such as people, systems, policies, *etc.* In addition, different types of insider threat incidents need to be considered (*e.g.*, sabotage, fraud, theft, *etc.*) for the aim of preventing a wide range of insider threat incidents.

#### The independence of system processes

A perfect prevention approach should prevent insider threats automatically and instantly, which is not found yet. In our review, we have noticed that some approaches require human interventions within their internal processes. For instance, the approach in Ragavan & Panda (2013) requires manual verifications to verify insider malicious operations on database data items. Also, the approach in Costante et al. (2016) requires a human factor to be included in the middle of framework operations. His/her task is to determine whether an alert raised by the framework belongs to a malicious act or not. Ideally, the decision of preventing an insider attack by an insider threat prevention system should occur automatically and independently. This is to avoid any slight delay that may be caused by a human intervention, which may leave an attack to occur causing massive damage for target assets.

#### The consistency and stability

To ensure that the results of evaluating a specific approach are reliable and consistent and over time, the approach should be validated with repeated experiments over different periods of time. Some of the reviewed approaches considered this factor in their experiments. For example, Eberz et al. (2016) conducted three sessions of experiments over various periods. The first session was performed, and after two weeks the second one was carried out. The third session was conducted after one hour of completing the previous one. Each session consisted of three experiments, and every experiment was repeated 5 times. The age factor of participants is also considered, which is distributed from 10 up to 50 years. The accuracy of the approach reached 92.2%. Thus, the authors showed confidence in the achieved results, as the factor of consistency and stability was confirmed.

The consistency of experimental results may also be influenced by other factors, especially when an approach is based on behavioral or physiological biometrics, such as in Eberz et al. (2016), Almehmadi & El-Khatib (2017) and Almehmadi (2018). We believe that deploying such approaches based on biometric characteristics (*e.g.*, eye motions, head micro-movements, and brain signals) can be influenced by other characteristics of the human body (*e.g.*, breathing, talking, yawning, nodding, *etc.*). So, further study needs to be done by insisting on the stability and consistency factors to establish more reliable solutions.

#### The coverage and scalability

The implementation of an insider prevention system is organizational-based, as the aim is to prevent malicious acts that could be carried out by employees of an organization. A medium-sized organization may have a large number of employees performing different types of tasks, and they may increase gradually. Thus, an insider threat prevention system should be scalable to handle the growing number of insiders as well as the tasks within an organization. Table 7 shows to which extent the reviewed approaches considered the number of insiders and tasks in their experiments.

**Table 7:** Number of insiders and resources handled by the insider threat prevention approaches.

Ref.	Focus	Count
Erdin et al. (2018)	Resources	4
O’Madadhain et al. (2005)	Insiders	250
Almehmadi & K. El-Khatib (2017)	Insiders	40
Babu & Bhanu (2015)	Insiders	11
Almehmadi (2018)	Insiders	30
Sibai & Menasce (2011)	Insiders	30
Chagarlamudi, Panda & Hu (2009)	SQL transactions	150
Ragavan & Panda (2013)	Write operations	111
Costante et al. (2016)	Insiders	100
Eberz et al. (2016)	Insiders	30

It has been noticed that some of the approaches highlighted the number of tasks/resources in their experiments, while the others emphasized the number of insiders that they handled. In Erdin et al. (2018), Chagarlamudi, Panda & Hu (2009) and Ragavan & Panda (2013), the focus was to evaluate the performance of an approach on the number of processed tasks, such as resource usages, SQL transactions, write operations, *etc.* On the other hand, the approaches in Almehmadi (2018), Babu & Bhanu (2015), Eberz et al. (2016), Almehmadi & El-Khatib (2017), Costante et al. (2016), Sibai & Menascé (2011) and Baracaldo, Palanisamy & Joshi (2019) focused on the number of handled insiders. Noticeably, we have observed that the largest number of insiders “250” have been addressed in Baracaldo, Palanisamy & Joshi (2019) compared to other approaches, whereas the least number of insiders “11” was handled in Babu & Bhanu (2015). The number of insiders, resources and operations that are handled by reviewed approaches are summarized in Table 7. It is observed that the approaches in Costante et al. (2016) and Baracaldo, Palanisamy & Joshi (2019) have been evaluated using 100 and 250 insiders respectively. Thus, finding a large-scale system to prevent insider threats remains a challenge, especially in ever-expanding organizations.

### Empirical aspects

**Figure 5:**
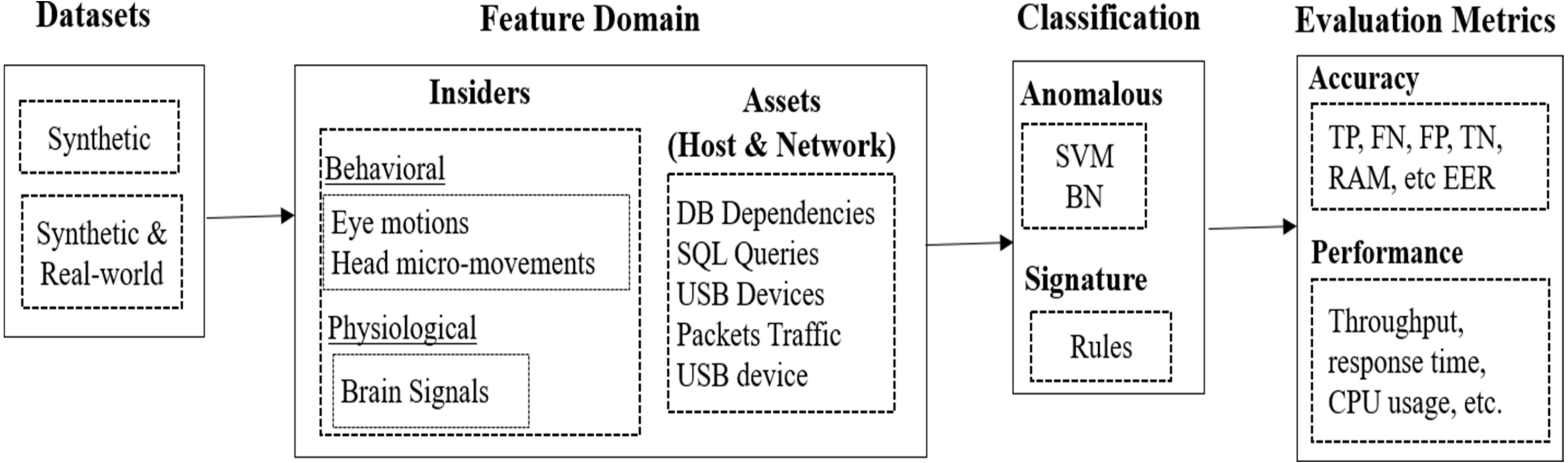
Empirical factors of the insider threat prevention approaches.

The appropriate description of experimental setup allows readers to understand the implemented approach very well. Moreover, interested researchers would be able to replicate an approach in a similar context for the sake of verification and improvement (Kitchenham et al., 2002). Re-implementing an approach cannot be achieved, if there is an inadequate explanation of its experimental settings. In this regard, we observed that the approaches in Erdin et al. (2018), Eberz et al. (2016), Almehmadi & El-Khatib (2017) and Almehmadi (2018) provided sufficient details that can enable researchers and practitioners to replicate them. Insider threat prevention is not a relatively mature research topic, so further work needs to be done to improve the existing works. In general, the empirical approaches contain several phases that need to be implemented in sequence (*e.g.*, data collection, feature extraction, classification, and presenting the results). In the coming sections we discuss who the reviewed approaches consider such aspects in a comparable manner. Figure 5 demonstrates the empirical aspects of reviewed approaches.

#### Datasets

Starting with the dataset collection aspect, various synthetic and real-world datasets are utilized for validating the reviewed approaches. If real-world datasets are commonly available in a research subject, it will be reflected positively in the advancement of solutions in that subject of research. However, researchers in the insider threat area of research are facing a challenge of obtaining real-world datasets due to privacy concerns. Many organizations are afraid of negative impacts that they may face, if they announce insider attack incidents that are executed by their employees (Roy Sarkar, 2010). Therefore, the scarcity of real-world datasets triggers some researchers to create synthetic datasets and make them available for the public. Table 8 presents the datasets that are utilized to validate the reviewed insider threat prevention approaches.

It is noticed that the work in Costante et al. (2016) and Baracaldo, Palanisamy & Joshi (2019) both synthetic and real-world datasets are employed. For instance, in Costante et al. (2016) a synthetic dataset was created by simulation on the healthcare management system (Gnu Health). A number of 30,490 SQL queries were collected over a period of 15 days. The authors made the created dataset available for researchers at Solidario (2020). With regard to real-world dataset, they got it from an Oracle database of a large IT company in the Netherlands. They kept it anonymous for privacy concerns. The dataset included 12,040,910 database transactions. In Baracaldo, Palanisamy & Joshi (2019), both synthetic and real-world datasets are combined. The dataset involves 5000 data items representing various types of database transactions. So, the approaches in Costante et al. (2016) and Baracaldo, Palanisamy & Joshi (2019) utilized both the synthetic and real-world datasets, and they made them public at Solidario (2020) and Baracaldo, Palanisamy & Joshi (2019), respectively. On the other hand, the work in Chagarlamudi, Panda & Hu (2009), Almehmadi & El-Khatib (2017), Eberz et al. (2016), Ragavan & Panda (2013), Erdin et al. (2018), Sibai & Menascé (2011) and Almehmadi (2018) created synthetic datasets, but they kept them private. Some approaches utilized datasets produced by others. For example, the approach in Babu & Bhanu (2015) was validated utilizing the CERT dataset (CERT and ExactData LLC, 2020). Such a dataset is well-known as it was used by several insider threat research studies (Roberts et al., 2016; Legg et al., 2017; Senator et al., 2013; Tuor et al., 2017; Gamachchi, Sun & Boztas, 2018).

**Table 8:** The utilized datasets for validateing the reveiwed approaches.

Ref.	Dataset	Availability
Chagarlamudi, Panda & Hu (2009)	Synthetic	Private
Almehmadi & K. El-Khatib (2017)	Synthetic	Private
Babu & Bhanu (2015)	Synthetic (CERT)	Public at [53]
Eberz et al. (2016)	Synthetic	Private
Ragavan & Panda (2013)	Synthetic	Private
Erdin et al. (2018)	Synthetic	Private
Costante et al. (2016)	Synthetic & Real-world	Public at [54]
Sibai & Menasce (2011)	Synthetic	Private
Almehmadi (2018)	Synthetic	Private
O’Madadhain et al. (2005)	Synthetic & Real-world	Public at [45]

The efforts of researchers for creating synthetic datasets and making them available online (*e.g.*, CERT dataset CERT and ExactData LLC, 2020), will facilitate and accelerate the progress of the insider threat area of research. We believe that creating synthetic datasets cannot replace real-world datasets, but complementing them as the synthetic datasets may not reflect actual motivations/intentions/behaviors of insiders in a real work environment. Furthermore, synthetic datasets, especially those created in-house, might be affected by subjective and biased surroundings. Although the availability of synthetic datasets (*e.g.*, CERT and ExactData LLC, 2020; Solidario, 2020; Baracaldo, Palanisamy & Joshi, 2019), there remains a gap to validate insider threat prevention approaches over real-world datasets.

#### Features domain

Datasets include diverse raw data that is captured while insiders interact with different types of assets, such as files, emails, websites, USB devices, *etc.* After that, observables are extracted from raw data as features (*e.g.*, logging/off outside working hours, sending to WikiLeaks, deleting backup files, installing malware, *etc.*) that can be utilized to prevent malicious acts of an insider. In an insider threat prevention system, vast amounts of data can be collected by numerous sensors distributed across an organization. Thus, selecting the most representative features from too high-dimensional data is the key to detect and prevent malicious acts more accurately. This section highlights various features of datasets that are employed by reviewed approaches. Table 9 classifies and summarizes the approaches, domain, and data features of reviewed articles.

**Table 9:** Approaches, domain and data features of the reviewed apparoches.

Ref.	Approach	Domain	Features
Chagarlamudi, Panda & Hu (2009)	Asset-based (Host)	Databases	DB Transactions
Almehmadi & K. El-Khatib (2017)	Biometric-based (Physiological)	Insiders	Brain signals
Babu & Bhanu (2015)	Biometric-based (Behavioral)	Insiders	Typing patterns
Eberz et al. (2016)	Biometric-based (Behavioral)	Insiders	Eyes motions
Murata (1989)	Asset-based (Host)	Databases	DB transactions, and dependencies
Erdin et al. (2018)	Asset-based (Host)	Computers	USB devices
Costante et al. (2016)	Asset-based (Host)	Databases	SQL queries
Sibai & Menasce (2011)	Asset-based (Network-based)	Network packets	Requests and Responses
Almehmadi (2018)	Biometric-based (Behavioral)	Insiders	Head motions
O’Madadhain et al. (2005)	Combined (Host and Network)	Geo-Social	Locations, devices and connections

As presented in Table 9, the insider threat prevention approaches are validated utilizing different data features extracted from various domains. In Chagarlamudi, Panda & Hu (2009), Ragavan & Panda (2013), Erdin et al. (2018), Costante et al. (2016) and Sibai & Menascé (2011) data features are extracted through asset-based (host and network domains). On the other hand, in Almehmadi & El-Khatib (2017), Babu & Bhanu (2015), Eberz et al. (2016) and Almehmadi (2018), data features are mined from an insider biometric domain: Physiological (brain signals) and Behavioral (typing patterns, eye and head motions).

Noticeably, the data features in Baracaldo, Palanisamy & Joshi (2019) were combined from different domains (Geo-Social). In such an approach, the experiments were conducted on 250 insiders, where the data features are selected from their geo-social context. They included diverse data features, such as devices used by insiders, insiders’ locations within their working environment, types of connections, *etc.* We believe that the combining of such geo-social features across the working environment of an insider, will provide rich information about possible suspicious acts that can assist decision makers to detect and prevent insider attacks proactively.

Nevertheless, through data features extraction process, some significant factors need to be considered in order to select the most accurate features associated with malicious acts of an insider (*e.g.*, external influences surrounding the acts of insiders, the dependent or independent features with respect to insider normal/malicious acts, and the stability of selected features overtime). In our review, we have observed that the study in Eberz et al. (2016) considered some of such factors while tracking eye motions of an insider to prevent masquerader attacks. For instance, the task-independent of collected features, the influences of high-dimensional feature sets, and the stability of data features on different conditions. Therefore, the highlighted features and relevant factors can give insights to extract the most reliable data features by future works.

#### Classification algorithms

Once datasets are collected, data features are extracted, the normal and malicious acts are classified utilizing different classifiers. The accuracy of an approach depends on selecting the proper classification algorithm (Azaria et al., 2014). This section illustrates the classification algorithms, statistical and matching methods that are employed by reviewed approaches as summarized in Table 10. It is noticed that the Support Vector Machine (SVM) classifier was utilized by three approaches (Almehmadi & El-Khatib, 2017; Babu & Bhanu, 2015) and (Eberz et al., 2016). Such a classifier is used widely in different classification problems, as it gives high performance results. For instance, in Almehmadi & El-Khatib (2017) the accuracy of the classification achieved 100%.

**Table 10:** The classification methods employed by insider threat prevention approaches.

Ref.	Classification method
Chagarlamudi, Panda & Hu (2009)	Signature matching
Almehmadi & K. El-Khatib (2017)	SVM
Babu & Bhanu (2015)	SVM
Eberz et al. (2016)	SVM
Ragavan & Panda (2013)	Signature matching & Anomalous modeling
Erdin et al. (2018)	Signature matching
Costante et al. (2016)	Signature matching & Anomalous modeling
Sibai & Menasce (2011)	Signature matching
Almehmadi (2018)	Statistical modeling
O’Madadhain et al. (2005)	Anomalous modeling

The approaches in Chagarlamudi, Panda & Hu (2009); Erdin et al. (2018); Sibai & Menascé (2011) utilized signature matching methods, where malicious acts are prevented by matching them with predefined threats. In Baracaldo, Palanisamy & Joshi (2019), an anomalous modeling was employed to prevent the insider malicious acts that can be deviated from normal ones. In Ragavan & Panda (2013) and Costante et al. (2016), both the signature matching and the anomalous modeling were implemented. However, several machine learning algorithms can be applied on the insider threat prevention subject (*e.g.*, SVM, Naïve Bayes, Decision Tree, K-Means Clustering, Random Forest, K-Nearest Neighbor (KNN), *etc.*). Most of them are openly available, such as on Weka (Hall et al., 2009) machine learning platform. Furthermore, Scikit-learn (Nelli, 2015), the most useful and robust library for supervised and unsupervised machine learning algorithms, can also be employed. It provides an efficient and wide range of tools for machine learning and statistical modeling (*e.g.*, regression, classification and clustering) *via* a consistent Application Programming Interface (API) in Python. Such free toolkits can be employed to develop and enhance more efficient systems.

#### Evaluation metrics

The clear demonstration of evaluation results for an insider threat prevention approach is highly significant. It provides assessment metrics to show the accuracy and performance of an approach and the significance of reported results. It has been observed that the reviewed approaches utilized various evaluation metrics, as summarized in Table 11. It is observed that the works in Erdin et al. (2018), Ragavan & Panda (2013) and Sibai & Menascé (2011) focused on assessing the performance of their approaches (*e.g.*, frequency, throughput, average response time and CPU utilization) rather than their accuracy in preventing malicious acts of insiders.

**Table 11:** The evaluation metrics of the reviewed approaches.

Metrics	Description	Ref.
FN	False Negative (FN) is the number of malicious acts that are not prevented by an approach.	Chagarlamudi, Panda & Hu (2009)
RAM	Risk Assessment Matrix (RAM) calculates the risk level for an asset with respect to malicious acts of an insider.	Almehmadi & K. El-Khatib (2017)
EER	Equal Error Rate (EER) is the rate of an intersection between False Acceptance Rate (FAR) and False Rejection Rate (FRR).	Babu & Bhanu (2015), Eberz et al. (2016)
Frequency and time	Determine the performance of the approach by calculating the frequency of validations and the time taken to address the threats.	Ragavan & Panda (2013)
Transferring time	The average time of transferring data from PC to USB device while preventing USB malicious code injections.	Erdin et al. (2018)
FP	False Positive (FP) is the number of legitimate activities of an insider that are counted as malicious ones.	Costante et al. (2016)
Performance measures	Determine the performance of the approach in terms of throughput, average response time, and CPU utilization.	Sibai & Menasce (2011)
Accuracy and acceptance rate	The accuracy rate of preventing malicious acts from insiders, and the acceptance rate of insiders for the measurements devices mounted on their heads.	Almehmadi (2018)
TP, FN, FP and TN	True Positive (TP) is the percentage of malicious acts prevented correctly. False Negative (FN) is the percentage of malicious acts that are not prevented. False Positive (FP) is the percentage of legitimate acts of an insider that are counted wrongly by an approach as malicious ones. True Negative (TN) is the percentage of legitimate acts that are classified correctly as legitimate.	O’Madadhain et al. (2005)

On the other hand, the other reviewed approaches focused on evaluating the accuracy for preventing insider malicious acts using different metrics. For example, the approaches in Almehmadi (2018) and Almehmadi & El-Khatib (2017) were evaluated utilizing the accuracy rate and risk assessment matrix, respectively. The approaches in Babu & Bhanu (2015) and Eberz et al. (2016) were evaluated using equal error rate, which is the intersection between the false acceptance rate and the false rejection rate.

With regard to the evaluation metrics, we believe that the TP, FN, FP and TN metrics are the best ones to assess the extent of how an approach is accurate in preventing insider malicious acts. These metrics are also known as a confusion matrix, which utilize several approaches (Chagarlamudi, Panda & Hu, 2009; Costante et al., 2016; Baracaldo, Palanisamy & Joshi, 2019). Table 12 shows a brief overview of the confusion matrix. Accordingly, an efficient insider threat prevention approach should minimize (FN and FP) and maximize (TP and TN). These metrics that are deduced from the confusion matrix are used commonly to evaluate several classification problems (Sokolova & Lapalme, 2009). Therefore, we recommend such metrics to be utilized for evaluating future insider threat prevention approaches.

**Table 12:** Confusion matrix (accuracy metrics) of the insider threat prevention approaches.

Action∖Reaction	Prevented	Not Prevented
Malicious act	True Positive (TP)	False Negative (FN)
Legitimate act	False Positive (FP)	True Negative (TN)

## Insider Risk Management Tools (IRMT)

An insider threat problem is a people centric issue that can come from users (employees or contractors) within an organization either maliciously, carelessly or negligently. The insider threat may happen in various forms (*e.g.*, fraud, theft, sabotage, *etc.*), which affect valuable assets of an organization causing severe damage to its reputation and business goals. Several monitoring and surveillance systems have emerged in the market for the aim of preventing and mitigating insider threats throughout organizations. In this section, we explore the commercial tools and products available in the industry defined as Insider Risk Management (IRM) solutions, which are designed to protect organizations from insider threat incidents. There are several IRM systems, so we give a brief view of the most well-known solutions (Erkan, Veriato, Proofpoint, and Teramind).

Ekran System^®^ (Ekran, 2022) is a solution that protects against insider threats on a global scale. It allows a company to keep track of the activity of its workers and contractors. Users’ activity on crucial endpoints, data, and configurations are monitored, recorded, and audited using the system platform. The system platform has a number of features that are used in a complicated alerting system (*e.g.*, session video records, anomaly detection, flags risky actions, real-time responses, *etc.*). The system includes a variety of toolsets for preventing insider threat incidents, which may be handled manually or automatically (*e.g.*, user alerting and blocking, activity termination, *etc.*). It also provides access management functions (*e.g.*, two-factor authentication, privileges and credential management, *etc.*). It’s built to meet security standards (*e.g.*, NIST, HIPAA, SWIFT, ISO, *etc.*) and it is ISO 27001 and ISO 9001 certified. It comes with a customizable licensing system, making it an all-in-one solution for implementing a solid security policy inside an organization.

Proofpoint^®^ (Proofpoint, 2022) is an IRM and Endpoint Data Leakage Prevention (DLP) solution. It protects against data loss and malicious activities that can be carried out by insiders whether maliciously or negligently. Proofpoint assists security teams for detecting and preventing insider data breaches by providing visibility, context, and analysis capabilities for incident investigations and response. Endpoint DLP is a subtype of IRM that focuses on identifying and preventing harmful user activity, whereas IRM focuses on monitoring and detecting hostile insiders. The system was built on a cloud platform which can be deployed, adapted and updated faster than on inside-organization tools.

Veriato^®^ (Veriato, 2022) Insider Risk Management & Employee Monitoring Solutions are a combined insider threat security platform. It is powered by machine learning and artificial intelligence. It integrates both User Activity Monitoring (UAM) and User Behavior Analytics (UBA) to provide a solid insider threat solution. The aim is to enable an organization for detecting and reacting to malicious acts quickly. It has several functions for monitoring emails, web browsing, chatting, document transferring, *etc.* Activities logs can be viewed with screenshots to verify that a positive threat is found with a fast response time.

Teramind^®^ (Teramind, 2022) is one of the most well-known insider threat solutions. It offers a monitoring capability of users’ behaviors using a user-centric security approach. It provides different functions for detecting malicious activity, improving users’ efficiency and productivity, and ensuring industry compliance. It enables real-time access to users’ activities to mitigate and prevent insider threat incidents. This is by providing functions for alerting, warnings, redirecting and blocking activities of malicious insiders. It also offers monitoring capability through a free Android app to secure the working place within an organization efficiently. Moreover, the system can be installed and deployed in a short time without users knowing which provides a full trust toward users making the work environment within an organization safe and more transparent.

## Research Challenges

This section presents some research challenges that need to be addressed within the insider threat prevention topic.

• *Ignorant Insiders*

An insider is a person who can access and use the systems and network of an organization in an authorized way. An insider attack may also be posed unintentionally by an ignorant insider who lacks the security awareness and knowledge making a severe threat to an organization’s IT infrastructure. For example, an ignorant insider can help an external adversary to control a node within an organization and extract credential information which can be easily re-programmed and replicated. This can enable the adversary to control the whole network of an organization and carry out various malicious acts. In Numan et al. (2020), several clone node detection schemes are presented which can be employed for preventing the threats that can be conducted due to uneducated or unsophisticated insiders. Furthermore, an ignorant insider can assist cyber criminals unconsciously to conduct botnet attacks within an organization network causing a massive scale of malicious acts (credentials leaks, data theft, send spams, DDoS attacks, *etc.*). Such an attack can be mitigated by employing an adaptive multi-layer botnet detection as it demonstrated an average accuracy of 98.7% (Khan et al., 2019).

• *Big Data Analytics*

An insider threat prevention system should deal with a huge amount of data that is coming from a wide range of sensors distributed within an organization (*e.g.*, computers, network tools, servers, *etc.*). The collected data are driven from diverse operating systems and protocols which need to be homogeneous in a central location for storage, viewing, and analysis. Thus, challenges arise while collecting and analyzing the insider threat prevention data, such as hardware faults, software bugs, and so on. The collection and analysis of data logs and system events for threat detection purposes have been a challenge in the information security community of research for decades. The traditional technologies are not suitable to support large-scale and long-term analytics for two reasons (Cardenas, Manadhata & Rajan, 2013): First, the collection and storage of huge amounts of data continuously are not feasible utilizing traditional infrastructures, so the collected data need to be deleted after a fixed retention period. Second, the analysis of large and unstructured datasets containing too many noisy features need to be cleaned, prepared, and analyzed efficiently. Blockchain is a new trend to enhance big data services due to its decentralization and security features (Zhang et al., 2021) and (Wang et al., 2021). So, several blockchain solutions for securing big data collection and storage, data analytics, and data privacy protection are reviewed in (Deepa et al., 2020). It also discusses different challenges and future directions which can drive research in the insider threat prevention area.

The new big data technologies (*e.g.*, the Hadoop and MapReduce ecosystems) provide a new trend of analyzing large-scale and heterogeneous datasets at unprecedented speeds and scales. These technologies are facilitating the storage, maintenance, and analysis of security information within an organization extremely. Thus, such technologies can be utilized in the area of insider threat prevention to efficiently process data for security analysis.

• *Cloud Computing*

In the last decades, cloud computing has attracted much attention in business, as it provides numerous computing functions (*e.g.*, Software-as-a-Service (SaaS), Platform-as-a-Service (PaaS) and Infrastructure-as-a-service (IaaS)). Cloud computing simplifies the access to a wide range of computing resources. The study in Zeadally et al. (2012) indicated that the Cloud Security Alliance reported the most significant threats for cloud computing and the malicious insider was listed among the top seven of them. In the cloud computing environment, it becomes difficult to manage security controls for providing highly distributed and mashup services (*e.g.*, Web API services), as an external action could be considered faulty as an authorized or unauthorized behind a firewall and an intrusion detection system of an organization. As illustrated above, the existing insider threat prevention systems yielded many false negative and false positive outcomes, therefore, much research needs to be done to prevent malicious insider actions, and allow benign insider actions within the cloud computing environment effectively. Recently, the malware in The Internet of Things (IoT) networking environment is one of the most serious security challenges. The IoT is a new technology which has been applied in different fields (Sitharthan et al., 2020). In Taheri et al. (2020), a federated learning-based architecture called (Fed-IoT) was proposed to detect Android malware applications in the Industrial IoT. This technique showed an improved accuracy rate in the protection of data privacy for Android mobile users with a percentage of 8% higher accuracy than the existing approaches. Moreover, the survey in Pham et al. (2021) reviewed the approaches of integration federated learning with IoT for the aim of securing resources and data management to provide safe and accurate protection models. Such techniques can be employed to detect possible Android malware attacks that can be injected by malicious insiders within an organization.

## Conclusion

Organizations are facing an increasing number of insider threats. As insiders have privileged access to the assets of an organization, preventing insider threats is a challenging problem. In this article, we reviewed the techniques and countermeasures that have been proposed to prevent insider attacks, in particular, we focused on approaches that are validated with empirical results.

First, we presented the huge amount of financial and reputational losses that are caused by real insider attack incidents. The implications of such losses emphasize the urgent need for effective insider threat prevention techniques.

Secondly, we proposed a classification model that categorizes the existing approaches into two main classes: biometric-based and asset-based. The biometric-based approaches are further classified into physiological, behavioral and physical, while the asset-based approaches are classified into host, network and combined. Such classification will provide a better understanding of the existing works, and highlight some gaps that need to be bridged to institute more holistic solutions.

Thirdly, the significant empirical factors of the reviewed approaches are discussed and compared in terms of (datasets, feature domains, classification algorithms and evaluation metrics). Theoretical aspects are also discussed in terms of (detection and prevention *vs* detection, behavioral *vs* physiological, simulating real-world situations, human factor interventions within automated processes of a solution, scalability of an approach, demonstrating experimental setting details, and the stability of obtained results over time). Thus, we deem that such factors are crucial and should be taken into consideration when developing and implementing insider threat prevention systems.

Finally, some challenges and research gaps were underscored. Recommendations were also highlighted to assist researchers for developing the novel terrain on the studied topic. In the future work, we aim to propose a comprehensive framework for preventing insider threats in large scale organizations. Several state-of-the-art technologies (*e.g.*, blockchain, IoT, cloud computing, machine and deep learning, *etc.*) will be integrated for the aim of devising an all-encompassing insider threat prevention framework.

## Additional Information and Declarations

### Data Availability

The following information was supplied regarding data availability: This work is a literature review.
